# Motor expertise and performance in sport-specific priming tasks: a systematic review and meta-analysis

**DOI:** 10.7717/peerj.11243

**Published:** 2021-04-13

**Authors:** Ruichen Jiang, Fei Xie, Anmin Li

**Affiliations:** 1 School of Psychology, Shanghai University of Sport, Shanghai, China; 2 School of Teacher Education, Anqing Normal University, Anqing, Anhui, China; 3 School of Foreign Languages, Anqing Normal University, Anqing, Anhui, China

**Keywords:** Motor expertise, Priming task, Sport, Systematic review, Meta-analysis

## Abstract

**Objective:**

The present study aimed to summarize findings relevant to the influence of motor expertise on performance in sport-specific priming tasks and to examine potential moderators of this effect.

**Methodology:**

Data were collected from the China National Knowledge Infrastructure (CNKI), PsychInfo, Medline, Google Scholar, Web of Science, Baidu Scholar and Sport Discus and Dissertation Abstracts Online databases from January 1999 to April 2020, supplemented by manual bibliographies and meeting minutes. Stata software was used to perform the meta-analysis. Study quality was evaluated systematically using the Newcastle-Ottawa scale (NOS). Standard mean differences (SMDs) with 95% CIs were calculated with a random-effects model. The Cochrane *Q* test and *I^2^* statistic were used to evaluate heterogeneity. Begg funnel plots and Egger tests were conducted to assess publication bias.

**Results:**

Nine articles (including 12 studies) were ultimately included in the meta-analysis. Significant heterogeneity was observed among these studies (*Q* = 44.42, *P* < 0.001, *I^2^* = 75.2%) according to random-effects modeling. The results showed an overall advantage in favor of motor experts in sport-specific priming tasks (SMD = −1.01, 95% CI [−1.41 to −0.61]). However, the magnitude of that effect was moderated by sport type (interceptive sports/independent sports) and prime stimulus type (subliminal stimulus/supraliminal stimulus). No publication bias was detected by the Begg and Egger tests.

**Conclusions:**

In general, compared with those of nonexperts, the responses of motor experts’ responses to a target stimulus are easier and faster when the prime and target stimuli are consistent. However, the magnitude of this effect is moderated by sport type and prime stimulus type.

## Introduction

The priming effect usually refers to the promotion effect of a first presented stimulus (prime stimulus) on a second immediately emerging stimulus (target stimulus) (Cetnarski et al., 2014). This effect mainly presents as a shorter reaction time for processing the target stimulus and, in neural activity, as decreased activation of specific brain regions involved in the processing of prime stimuli (Schacter, Dobbins & Schnyer, 2004).

In sports practice, the priming effect is a common phenomenon and may reflect experts’ cognitive advantages (Hung et al., 2004). Increasing evidence suggests that motor experts not only have stronger athletic ability but also show advantages in the cognitive process (i.e., perception, anticipation and decision-making) (Yarrow, Brown & Krakauer, 2009; Nakata et al., 2010). In sports practice, motor experts need to extract effective cues from the complex environment as quickly as possible and predict the result of future movements (Mori & Shimada, 2013; Müller & Abernethy, 2012). Such prior representation of the relevant movement pattern structure enables faster responses and improved accuracy (Dehaene et al., 1998). Studies of visual search and perceptual prediction also suggest that the processing of a sequence consisting of successive stimuli is an important part of motion perception and executive control (Güldenpenning et al., 2013).

Expert/nonexpert differences in sport-specific priming effects can be interpreted using the theory of event coding (TEC) (Hommel et al., 2001). According to the TEC, perceived events (perceptions) and to-be-generated events (actions) are commonly coded according to an information structure model (i.e., independent movements, background information) and stored in experts’ long term memory. This model can be directly activated by the prime stimuli through top-down processing (Mann et al., 2007). More specifically, motor experts seem to benefit from the process of prime stimuli, which may be implicitly learned during regular training and competition (Güldenpenning et al., 2013). As a result, increasing attention has been given to research on the promotion of sport performance through priming task training (MacNamara, Button & Collins, 2010).

While many studies support the efficacy of priming, there is a paucity of research examining the priming effect on skilled motor behavior and the underlying processes that mediate any observed effects. In describing possible moderating variables of the priming effect in sport-specific tasks, we focus on variables identified in past studies. Some scholars have suggested that sport type is a potential moderator of the training-cognition relationship (Voss et al., 2010). Sports can be classified as interceptive (or coactive), independent (or propulsive), or strategic (or interactive) to determine the effect of sport type on expert/nonexpert comparisons (Mann et al., 2007). Athletes in different sports have different sources of cues in their perceptual responses, and endogenous cues are more beneficial than exogenous cues (stimulative or inhibitory) for improving response time (Voss et al., 2010).

In addition, the prime stimuli in the cue-target paradigm can be classified as subliminal stimuli and supraliminal stimuli according to their visibility (Draine & Greenwald, 1998; Skandrani-Marzouki & Marzouki, 2010). The presentation time of a subliminal prime is very short (≤50 ms), and the prime is masked. Furthermore, the visibility of the prime should be tested separately (Kiesel, Kunde & Hoffmann, 2007). Whether the prime stimuli is visible or not is also a significant moderator of the effect sizes in priming tasks (Bussche, Noortgate & Reynvoet, 2009). Subliminal prime stimuli and supraliminal prime stimuli lead to different priming effects in athletes (Meng et al., 2019). Therefore, the priming effects can also be categorized as subliminal priming and supraliminal priming. The stimulus-response association is activated at the unconscious level when subliminal priming occurs (Cetnarski et al., 2014). Motor experts, especially those who participate in open-skill sports (i.e., table tennis, tennis and badminton), tend to make unconscious motor responses to a given situation rather than consciously processing changes in the external environment (Meng et al., 2019).

Given the diverse approaches for examining expert/nonexpert differences in the priming effect put forth in the literature, our aims were twofold. First, we aimed to determine the overall effect of motor expertise on performance in sport-specific priming tasks. Perceptual-cognitive differences between motor experts and nonexperts in the priming effect have been confirmed using sport-specific stimuli, but prior studies only reported expert/nonexpert differences in related indicators (including reaction time, accuracy, etc.), and few studies have focused on the overall effect size. Second, if sufficient research reports could be obtained, we aimed to focus on subgroups, including sport type (interceptive sports or independent sports) and type of prime stimulus (subliminal stimuli or supraliminal stimuli). We tried to determine the extent to which priming effects are differentiated between different types of sports or prime stimuli. Accordingly, the present meta-analysis conducted systematic and quantitative analyses of empirical studies on the priming effect in sport from a macro perspective to obtain the overall priming effect value of motor expertise within the cue-target paradigm.

Based on the conclusions of previous studies, the following hypotheses are proposed: (1) experts exhibit faster response times in the priming task when the prime and target stimuli are consistent; and (2) based on the two moderator variables set in this meta-analysis, sport type moderates the expertise relationship for response time, and the cognitive advantage displayed by professional athletes varies with the type of prime stimuli.

## Methods

### Literature search

An exhaustive study retrieval was performed in an effort to locate all relevant studies, including research in China National Knowledge Infrastructure (CNKI), PsychInfo, Medline, Google Scholar, Web of Science (WOS), Baidu Scholar and Sport Discus and Dissertation Abstracts Online, using a combination of the following words: *sports, athletes, sports experts, expertise, physical exercise, physical activity, priming, cue-target, anticipation, information processing, overview or meta-analysis*. All the review articles and research studies obtained were examined by one investigator, followed by a manual search of the following journals: *Psychology of Sport & Exercise*, *British Journal of Sports Medicine*, *Journal of Sports Sciences*, *Cognition*, *Motor Control* and *International Journal of Sport Psychology*. Finally, we obtained unpublished data using emails to communicate with experts specializing in sport cognition.

### Eligibility and exclusion criteria

#### Eligibility criteria

The primary aim of this meta-analysis was to investigate whether motor experts respond more quickly than nonexperts when the cue and target stimulus are consistent in sport-specific priming tasks. In this regard, the included studies were required to meet the following criteria:

1. Study type: The included studies had to be cross-sectional studies with complete data. The research content and sample had to be reported in detail, as well as the mean values and standard deviations (or standard errors).
2. Study paradigm: The experimental paradigms used had to all be cue-target paradigms. The subjects had to respond to the target stimulus, and their response time had to be recorded.
3. Study population: Studies had to compare the priming effects of motor experts vs. a nonexpert group (novices). Included studies were required to recruit athletes identifiable as high-performing elite (i.e., competing at the highest level in their sport). The nonexpert group participants were referred to individuals not involved regularly in the relevant activity of the motor experts. The average ages of these two groups were similar.
4. Sports events: Sports were defined as activities that include physical activities and games to enhance and improve the physical skills of an individual. In addition, previous studies have shown the cognitive benefits of aerobic fitness (Hillman, Erickson & Kramer, 2008); thus, it was necessary to exclude those events that do not require running or jumping movement (e.g., golf, shooting) to minimize potential confusion of individual differences.
5. Outcome variable: The dependent variable was the response time to the target stimulus.

#### Exclusion criteria

**Figure 1:**
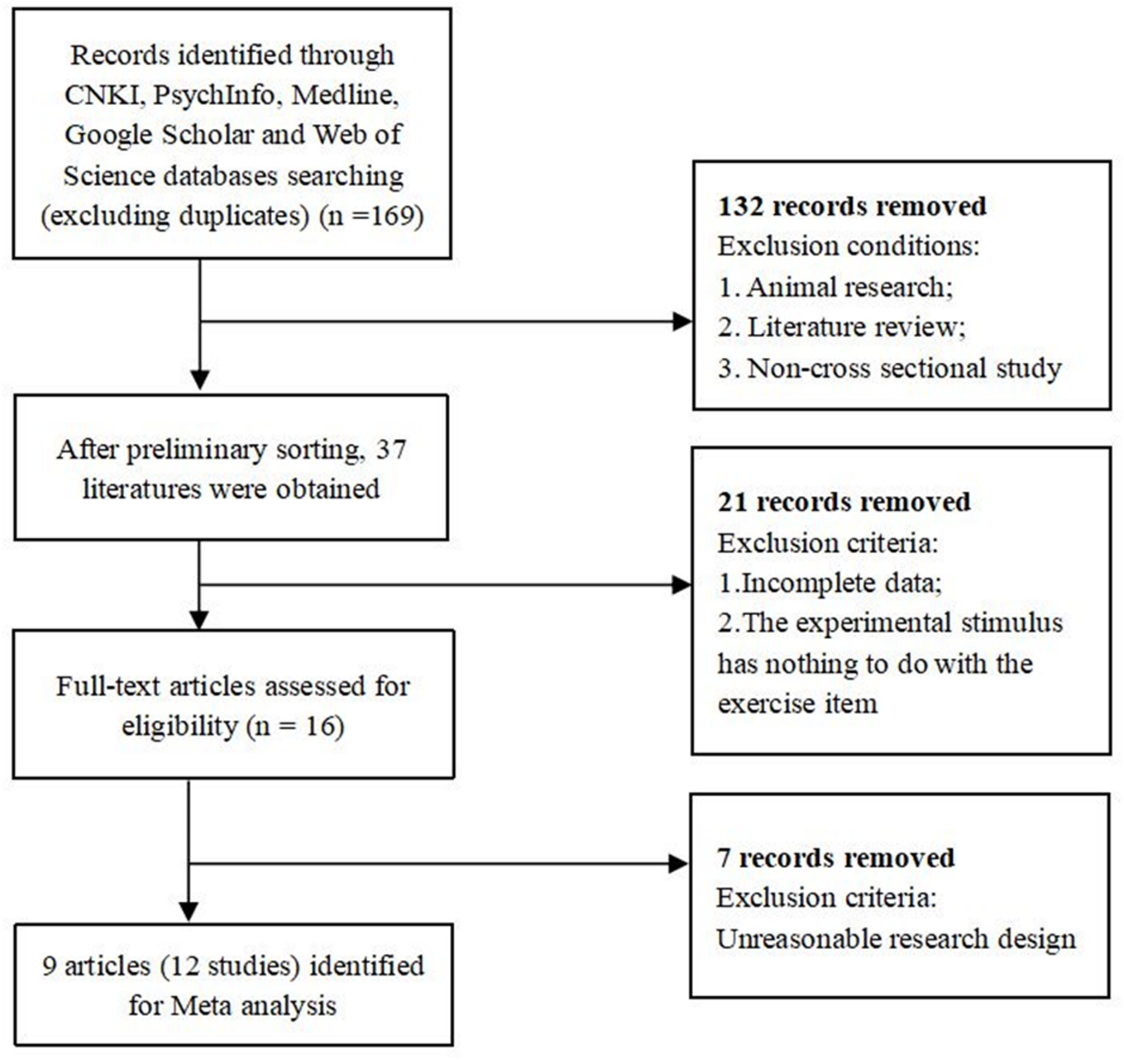
Flow diagram of study selection for meta-analysis.

The exclusion criteria were as follows: (1) review literature or nonempirical literature; (2) unavailable full text; (3) duplicate publications or publications based on the same batch of data; (4) incomplete research results and missing test data of motor expert groups and nonexpert groups (such as missing information on the standard deviations or standard errors); (5) research paradigms other than the cue-target paradigm; and (6) no connection between the stimulus material in the study and the sport item. The search strategy was performed by one investigator (Jiang), and the search process is summarized in Fig. 1.

### Study selection

To determine whether the inclusion criteria had been satisfied, two researchers (Li and Xie) were assigned to assess the obtained articles independently. Discrepancies in the evaluation of the articles were discussed until a consensus was reached. The investigators attempted to contact the corresponding author by email if the methods or results were not clearly described.

### Quality evaluation

The methodological quality of each study, including participant selection, control of confounding variables and the final outcome variables, was assessed independently by two researchers (Li and Xie) using an adapted version of the Newcastle-Ottawa scale (NOS), which contains eight items and is commonly used in cross-sectional studies (Oremus et al., 2012). The total score for all items was used to evaluate the quality of each study (i.e., >7 = high quality, 5–7 = moderate quality, <5 = low quality). Discrepancies were discussed, and if no consensus could be reached, a third researcher (Jiang) was invited to make the final decision. Studies that were rated as moderate quality or higher were eligible for the meta-analysis.

### Coding of variables

The following two factors mentioned in the introduction were included as moderators in the present meta-analysis. (1) Type of sport: In this meta-analysis, sports were classified as interceptive (or coactive), independent (or propulsive), or strategic (or interactive or invasive) to determine the effect of sport type on expert/nonexpert comparisons (Mann et al., 2007). A sport classified as independent included characteristics such as being closed, self-paced, and aiming at a target (e.g., gymnastics, rock climbing, and swimming). (2) Type of prime stimulus: Since subliminal prime stimuli and supraliminal prime stimuli lead to different priming effects (Meng et al., 2019), whether the prime is visible or not was also considered as a significant moderator of the effect sizes in priming tasks (Bussche, Noortgate & Reynvoet, 2009). Therefore, whether there is a general difference in effect size caused by different types of prime stimuli in the priming paradigm was distinguished. The generation of the effect value was based on independent samples, and each independent sample was coded once. If a study contained multiple independent samples, multiple coding was performed accordingly.

### Statistical analysis

Stata software version 12.0 (Stata, Inc., College Station, TX, USA) was used for data processing in this meta-analysis to calculate the effect size (ES) and standard mean difference (SMD) to reflect the effect indexes. Cohen’s *d* (Cohen, 1988) was used as an effect of the estimated effect size. The calculation method of *d* value was the same as the SMD. The formula is *d* = (M1–M2)/SD, in which M1 represents the average value of the expert group and refers to the average response time of the motor experts included in the meta-analysis; M2 represents the average value of the nonexpert group and refers to the average response time to the target stimulus of the nonexpert group in this meta-analysis; SD refers to the joint standard deviation of the motor expert and nonexpert groups. *P* < 0.05 was considered statistically significant. The pooled SMD was the final statistical index of the meta-analysis, and the heterogeneity of each study was checked before merging. Values of 0.20, 0.50, and 0.80 represent small, medium, and large effect size estimates, respectively (Cohen, 1988).

Statistical heterogeneity was evaluated using the chi-square-based *Q* statistic test, which ranges from 0% to 100% (*I*^*2*^ < 25%, no heterogeneity; *I*^*2*^ = 25–50%, moderate heterogeneity; *I*^*2*^ = 50–75%, large heterogeneity; and *I*^*2*^ > 75%, extreme heterogeneity) (Higgins et al., 2003). We performed analysis using fixed-effects models if the *Q*-test of heterogeneity was considered not significant; otherwise, random-effects models were used (Rebecca & Nan, 2015). Subgroup analysis and sensitivity analysis were then used to explore the source of the heterogeneity when heterogeneity remained (Lau, Ioannidis & Schmid, 1997). Begg funnel plots and Egger tests were conducted to assess potential publication bias (Peters et al., 2006).

## Results

### Characteristics of the subjects and studies

The included studies were published between 2009 and 2019. Of the 16 articles fulfilling the inclusion criteria, data from nine articles (12 studies) were available for final statistical analysis, and the other seven references were excluded because the study design did not meet the meta-analysis requirements. The pooled data yielded a total of 246 motor experts (48.8%) and 258 nonexperts (51.2%). Across all 12 studies, the participants’ age at the time of assessment ranged from 13 to 27 years (based on articles that reported the subjects’ ages). There was no statistically significant difference in the mean ages of motor experts and nonexperts (*P* > 0.05). The participant characteristics are reported in Table 1. Most of the studies were from peer-reviewed journal articles (80%), and the rest were from master’s papers. Five studies were from human kinesiology laboratories, and the others were from psychology laboratories. Nine of the included studies had NOS scores of high quality (≥7 points), and 3 had NOS scores of moderate quality (5–7 points).

**Table 1:** Literature coding results of meta-analysis.

Authors/Year	Type (sports)	*N* (e/c)	Age (e/c)	Type (PS)	Test material	Score	SE
Claire Calmels/2018	Independent	12/12	13.7/13.5	Subliminal	Picture	RT	−3.16
Fanying Meng/2019	Interceptive	42/42	20.27/20.55	Subliminal	Picture	RT	−1.18
Iris Gu¨ldenpenning/2011	Independent	16/16	24.1/22.3	Subliminal	Picture	RT	−0.61
Chenxi Jin/2015	Interceptive	19/23	19.89/20.55	Subliminal	Picture	RT	−0.94
Chun-Hao Wang/2017	Interceptive	16/16	20.56/20.56	Supraliminal	Picture	RT	−0.76
Chun-Hao Wang/2017	Interceptive	16/16	20.56/20.56	Supraliminal	Picture	RT	−0.81
Bettina E. Bläsing/2014	Independent	14/18	27.0/24.0	Supraliminal	Picture	RT	0.26
Chun-Hao Wang/2015	Interceptive	12/16	20.58/19.07	Supraliminal	Picture	RT	−0.82
Ai-hua Yang/2009	Interceptive	15/15	18–22/18–22	Supraliminal	Picture	RT	−1.12
Claire Calmels/2018	Independent	12/12	13.7/13.5	Subliminal	Picture	RT	−3.5
Fanying Meng/2019	Interceptive	42/42	20.27/20.55	Supraliminal	Picture	RT	−0.88
Shao-yi Zhang/2017	Interceptive	30/30	19–24/19–24	Supraliminal	Picture	RT	−0.44

**Note:**

N(c/e) = Number of experts/Number of control participants; Age (e/c) = Average age of the experts/Average age of the nonexperts; Type (PS) = Type of prime stimulus; SE = Effect size; RT = Reaction time.

### Meta-analysis results

**Figure 2:**
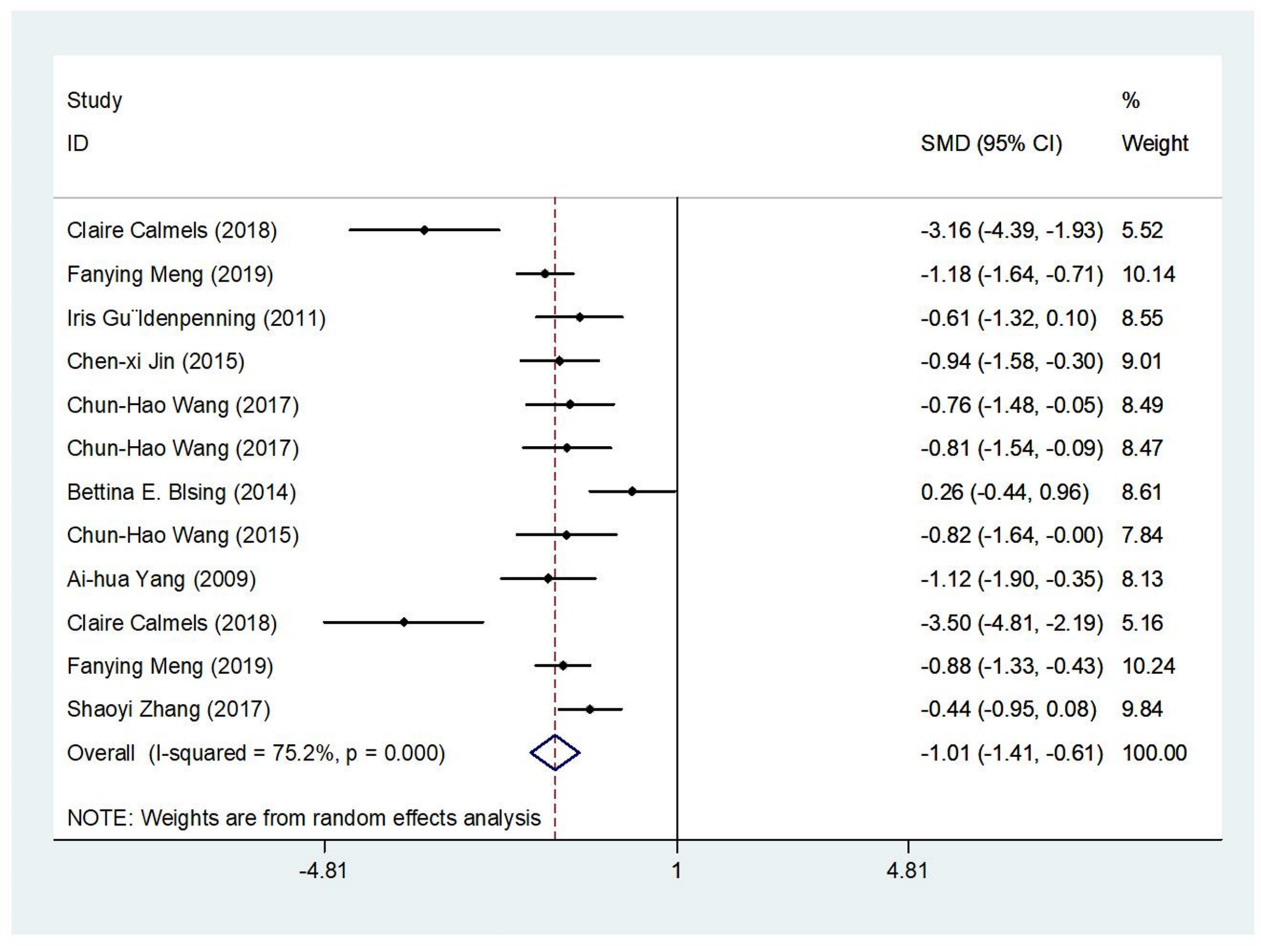
Results of random effects analysis. This graph is a forest plot of effect sizes as a function of authors and year of publication for the studies entered in the present analysis.

Meta-analysis was performed using the results from the 12 studies that fulfilled the inclusion criteria. Significant heterogeneity was detected (*Q* = 250.85, *P* < 0.001, *I*^*2*^ = 76%). Therefore, the pooled effect size was estimated with a random-effects model in order to provide a more conservative estimate of the effect sizes. Figure 2 shows the results of the random-effects model after the operation. The effect size after merging was −1.01, 95% CI [−1.41 to −0.61], and the result of the combined effect quantity hypothesis test was *Z* = 4.99, *P* < 0.001. The confidence interval of the SMD fell to the left of the equivalent line, indicating that the response time to the target stimulus of the expert group was significantly less than that of the nonexperts, thus validating research Hypothesis 1.

### Subgroup analyses

Subgroup analyses using a random-effects model were conducted to observe whether the two variables sport type and prime stimulus type explained the heterogeneity and contributed to the mean effect sizes. Of the literature included in this meta-analysis, the sport type was independent sport in four studies and interceptive sport in the remaining eight studies. The results of the subgroup analysis based on sport type showed that there were significant expert/nonexpert differences in the priming effect value for interceptive sports (SMD = −0.87, 95% CI [−1.08 to −0.66]; *P*(SMD) < 0.001), and no heterogeneity was reported (*I*^*2*^ = 0, *P* = 0.66). By contrast, no significant expert/nonexpert differences were found in the priming effect value for independent sports (SMD = −1.67, 95% CI [−3.35 to 0]; *P*(SMD) = 0.051), and extreme heterogeneity was reported (*I*^*2*^ = 92.4%, *P* < 0.001) (see Table 2). These results indicated that the priming effect of the motor expert group was significantly higher than that of the nonexpert group in interceptive sports. In addition, the type of prime stimulus was used as a basis for grouping. Among the 12 studies, 5 used subliminal prime stimuli, and 7 used supraliminal prime. The results of the subgroup analysis based on the prime stimulus type are shown in Table 2. Significant expert/nonexpert differences were found in the priming effect value for subliminal prime stimuli (SMD = −0.99, 95% CI [−1.32 to −0.66]; *P*(SMD) < 0.001), and no heterogeneity was reported (*I*^*2*^ = 0, *P* = 0.419), indicating that the priming effect value of the motor expert group was significantly higher than that of the nonexpert group when the prime stimuli were presented under a threshold. Significant expert/nonexpert differences were also found in the priming effect value for supraliminal prime stimuli (SMD = −1.09, 95% CI [−1.65 to −0.54]; *P*(SMD) < 0.001); however, extreme heterogeneity was reported (*I*^*2*^ = 81%, *P* < 0.001). Therefore, the results of the subgroup analysis validated research Hypothesis 2.

**Table 2:** Subgroups analysis: type of exercise and type of PS.

Moderator		Number of studies	SMD (95% CI)	*I*^2^ (%)	*P*	*P* (SMD)
Type of exercise	Interceptive	8	−0.87 [−1.08 to −0.66]	0	0.660	<0.001
	Independent	4	−1.67 [−3.35 to 0]	92.4	<0.001	0.051
Type of prime stimulus	Subliminal	3	−0.99 [−1.32 to −0.66]	0	0.419	<0.001
	Supraliminal	9	−1.09 [−1.65 to −0.54]	81.0	<0.001	<0.001

**Note:**

PS = prime stimuli.

### Assessing the risk of bias

**Figure 3:**
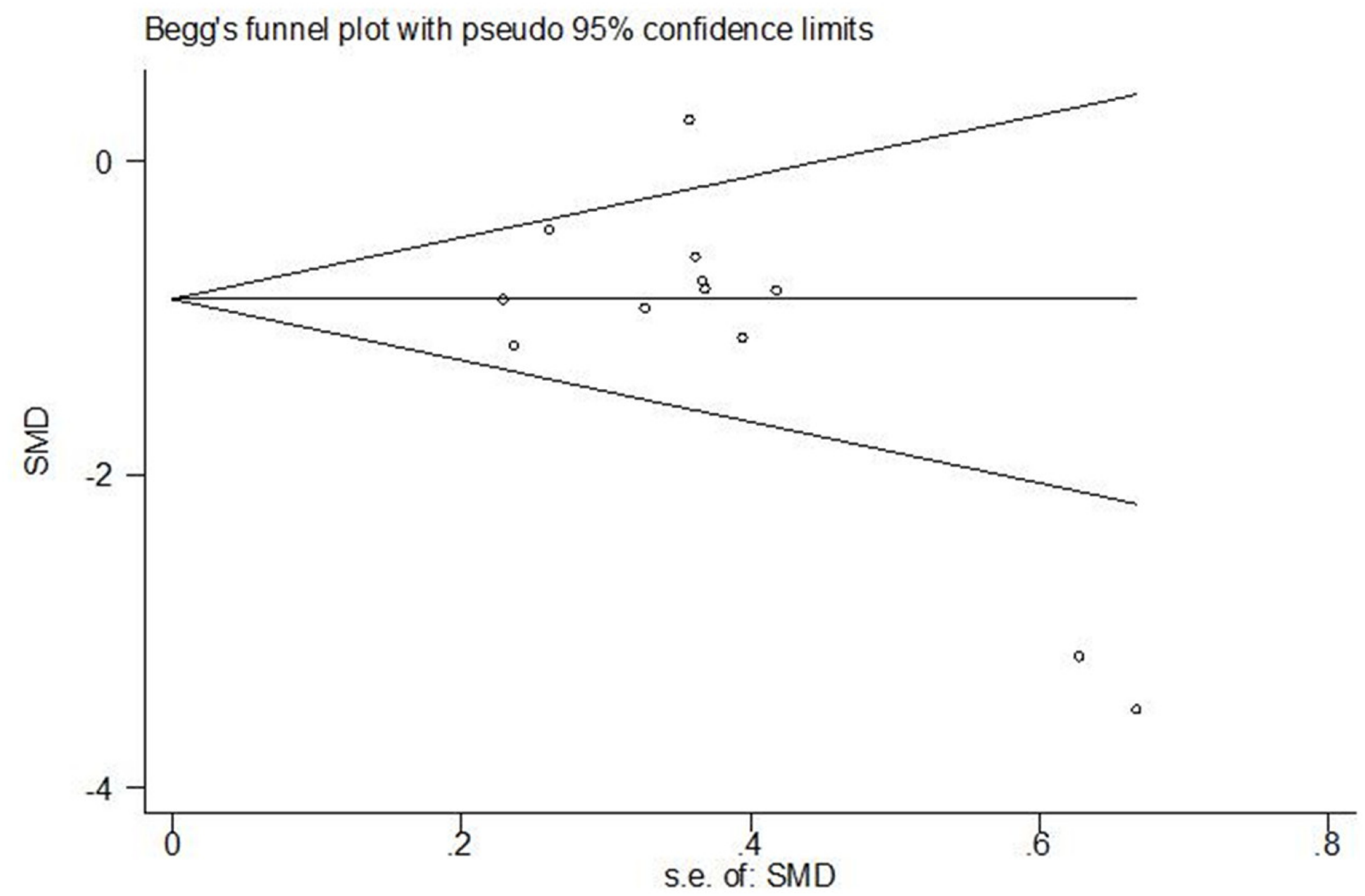
Begg’s funnel plot for publication bias. Circles represent individual studies.

The credibility of a meta-analysis depends on publication bias. A funnel plot was performed to explore the priming effect differences between motor experts and nonexperts. Most of the studies were concentrated at the top of the plot, and only a few studies deviated far away (Fig. 3). Additionally, we did not find evidence of publication bias in the Egger test (*P* = 0.093 > 0.05) or Begg test (*P* = 0.115 > 0.05). Thus, no significant publication bias existed in this meta-analysis.

### Sensitivity analyses

**Figure 4:**
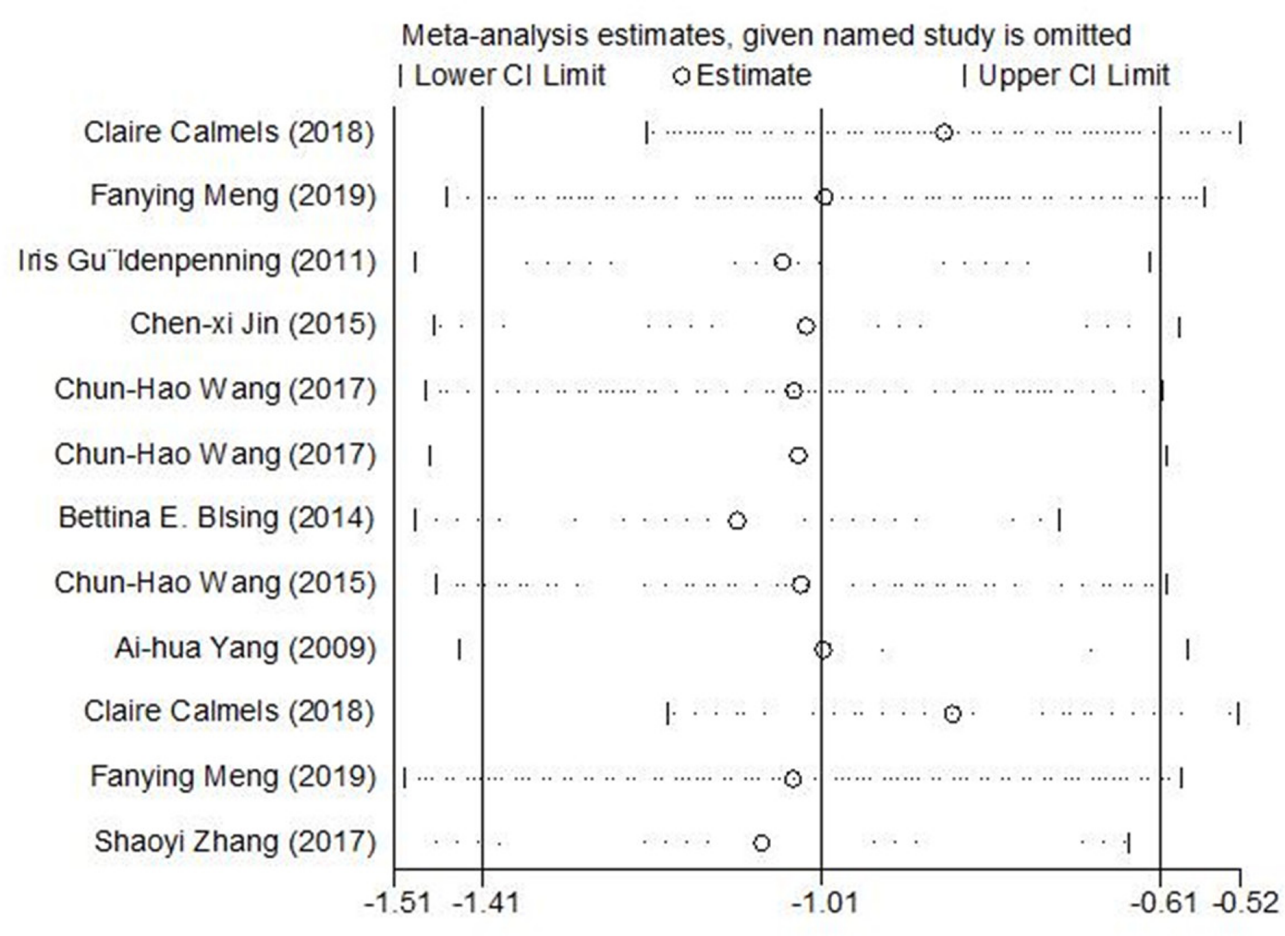
Sensitivity analyses by excluding one study at a time.

In view of the significant heterogeneities in the results, a sensitivity analysis of the 12 included studies was conducted. Figure 4 shows the pooled SEs and 95% CIs of the sensitivity analysis. The omission of any one study had no significant effect on the final result, indicating that the meta-analysis results were relatively reliable and robust.

## Discussion

This meta-analysis quantitatively examined the relationship between motor expertise and performance in sport-specific priming tasks. We were able to aggregate the effects across different studies despite the existence of heterogeneity. Consistent with previous studies (Schütz-Bosbach & Prinz, 2007; Dehaene et al., 1998), the average effect was still statistically beneficial for motor experts (Fig. 2), and no publication bias was found. A sensitivity analysis was also conducted to support the reliability and robustness of the main result, which suggested that motor experts show higher perceptual sensitivity to sport-specific stimuli than nonexperts and that images presented at the prime stage activate behavioral preparation related to the response stage, thereby affecting the response to the same or similar target stimuli (McPherson, 2000; Güldenpenning et al., 2011). These observations are perfectly in line with the TEC (Hommel et al., 2001). According to the TEC, experts’ attention is easily directed to the most important features of stimuli automatically, which allows rapid and effective pattern recognition and thus an accurate response to the target stimulus. This specific ability not only reduces the complexity of the environment but also enables experts to successfully deal with stimuli with limited cognitive resources. Novices lack this special knowledge structure to guide their perception; consequently, they are not easily affected by sport-specific prime stimuli and respond more slowly.

The results of the subgroup analysis indicated that sport type is a significant moderating variable of the expertise relationship for response time, suggesting that interceptive sport athletes show a statistically significant priming effect in sport-specific tasks. These findings are similar to those of previous studies in which athletes in interceptive sports showed the greatest benefit in terms of processing speed (Davids et al., 2002; Mann et al., 2007). However, in independent sports, no expert/nonexpert differences in the combined priming effect were reported, and the heterogeneity between studies was extreme. One potential explanation of these results is that an important characteristic of interceptive sports is that players need to respond to emergencies, make quick position changes and make decisions on the court (Ozmen & Aydogmus, 2016). For example, in a competitive ball game, the speed of the ball may reach the limit of an individual’s visual ability; players need to perceive the ball and its movement within a few milliseconds and then hit it back with accuracy (Sean & Bruce, 2012). Therefore, athletes who have been trained in interceptive sports with very tight time and space constraints have a better ability to extract the most valuable information and respond faster than novices (Güldenpenning et al., 2013), and their predictive ability might also be better developed (Mori & Shimada, 2013).

The prime stimulus type was also a significant moderating variable of the expertise relationship for proceeding time in sport-specific priming tasks. Significant expert/nonexpert differences and no heterogeneity were reported when the prime was subliminal, indicating that motor experts are more sensitive to subliminal stimuli. These results are consistent with previous research (Meng et al., 2019) and can be interpreted according to the two-stage process put forward by Kibele (2006) based on the TEC. Associations between perception and action are established unconsciously when subliminal prime stimuli appear, and the response is preactivated in the first stage. The second stage is performance. Under the condition of prime-target consistency, participants are able to process the prime stimuli in a goal-oriented manner after presentation of the rapid prime stimuli and complete the relevant action sequence in a coordinated and orderly manner, resulting in a faster response to the target stimulus (Radlo et al., 2001). For studies using supraliminal prime stimuli, significant expert/nonexpert differences in reaction time were reported with extreme heterogeneity, indicating that the conscious priming task may be too complex to distinguish experts from nonexperts.

Several limitations should be acknowledged in our meta-analysis. First, all of the included studies were based on a cross-sectional study design. Whether these experts acquire specific cognitive skills as a result of experiential learning remains to be resolved. A randomized controlled design could be adopted to take cognitive differences as a function of empirical variables (including the number of years of training, sport type, training intensity, etc.), and a comprehensive test could be conducted on a wide range of cognitive abilities to better reveal the relationship between motor expertise and cognitive development. Second, our findings reflect only behavioral differences between different groups. However, the reason for the better performance of experts than nonexperts in the priming task lies in differences in brain structure and function (Smith, 2016; Wright et al., 2011); therefore, in addition to summarizing the differences in performance of motor experts and nonexperts on priming tasks, future studies should seek to reveal the internal brain mechanisms.

In general, this meta-analysis shows that studies of the priming effect related to sport have demonstrated that the motion-cognition relationship is an outcome variable of training. The motion-cognition relationship deserves a place in the expanding knowledge of how exercise training affects the acquisition of basic cognitive abilities. In addition, the two important factors of sport type and prime stimulus type, which significantly moderate the relationship between motor expertise and perceptual skill, should be used to guide future research on expertise. In confrontational sports training, subliminal priming task maybe a good way to promote the athletes’ sport performance.

## Supplemental Information

10.7717/peerj.11243/supp-1 Supplemental Information 1 PRISMA checklist.

10.7717/peerj.11243/supp-2 Supplemental Information 2 Systematic Review and/or Meta-Analysis Rationale.

10.7717/peerj.11243/supp-3 Supplemental Information 3 The strengths of this article.

## Additional Information and Declarations

### Data Availability

The following information was supplied regarding data availability: The data are available in Table 1.
