# Supplementary material for: Motor expertise and performance in sport-specific priming tasks: a systematic review and meta-analysis
Source: PeerJ. 2021 Apr 13;9:e11243. doi: 10.7717/peerj.11243 (PMC8051356; doi:10.7717/peerj.11243)
Supplement: Supplemental Information 2 [file peerj-09-11243-s002.docx]

**Systematic Review and/or Meta-Analysis Rationale**

More and more evidences suggested that motor experts not only have stronger athletic ability, but also show advantages in the cognitive process (ie., perception, anticipation and decision-making). Perceptual-cognitive differences between motor experts and novices on the priming effect using stimulus related to sport have been confirmed, and this task could direct the experts' attention to the most important features of stimulus automatically, allowing rapid and effective pattern recognition and thus making the accurate response to the target stimulus. But prior studies only reported differences between experts and novices in related indicators (such as reaction time, accuracy, etc.), there were few studies which focused on the overall effect size. More specifically, if sufficient research reports could be obtained, the study will also focus on the situation of each subgroup, such as exercise type (interceptive sport or independent sport) and type of prime stimulus (subliminal stimuli or supraliminal stimuli). Thus we also sought to determine the extent to which priming effect discriminates between different types of sports or prime stimulus.
