# Supplementary material for: Motor expertise and performance in sport-specific priming tasks: a systematic review and meta-analysis
Source: PeerJ. 2021 Apr 13;9:e11243. doi: 10.7717/peerj.11243 (PMC8051356; doi:10.7717/peerj.11243)
Supplement: Supplemental Information 3 [file peerj-09-11243-s003.docx]

***The strengths of this article***

While many studies support the efficacy of priming in the aforementioned behavioral categories, there is a paucity of research examining the effect of priming on skilled motor behavior and the underlying processes that mediate any observed effects, and no systematic review or meta-analysis of priming effects in sport filed was found. This study reviewed the researches about priming effect in the sports field quantitatively. In general, compared to the novices, the motor experts’ response to the target stimulus will be easier and faster when the prime and target stimulus were consistent. This study again verified the relationship between motor expertise and perceptual advantage from a microscopic perspective。
